# Cognitive Vulnerability in Aging May Be Modulated by Education and Reserve in Healthy People

**DOI:** 10.3389/fnagi.2017.00340

**Published:** 2017-10-24

**Authors:** María D. Roldán-Tapia, Rosa Cánovas, Irene León, Juan García-Garcia

**Affiliations:** Department of Psychology, University of Almería, Almería, Spain

**Keywords:** well-aging, educational attainment, cognitive reserve, brain compensation, neuroplasticity, cognitive domains

## Abstract

Aging is related to a deterioration of cognitive performance and to multiple alterations in the brain. Even before the beginning of a noticeable cognitive decline, the framework which holds cognitive function experiences these alterations. From a system-vulnerability point of view of cognition, the deterioration associated with age would be the collection of repercussions during a life. Brain function and structure are modified in a multidimensional way, which could concern different aspects like structural integrity, functional activity, connectivity, or glucose metabolism. From this point of view, the effects of aging could affect the most brain systems and their functional activity. In this study, we analyze the functional development of three cognitive domains in relation to aging, educational level, and cognitive reserve (CR). A total of 172 healthy subjects were divided into two age groups (young and old), and completed a battery of classic neuropsychological tests. The tests were organized and analyzed according to three cognitive domains: working memory and flexibility, visuoconstructive functions, and declarative memory. Subjects also completed a questionnaire on CR. Results showed that the performance in all cognitive domains decreased with age. In particular, tests related to working memory, flexibility, and visuoconstructive abilities were influenced by age. Nevertheless, this effect was attenuated by effects of education, mainly in visuoconstructive domain. Surprisingly, visual as well as verbal memory tests were not affected either by aging, education, or CR. Brain plasticity plays a prominent role in the aging process, but, as other studies have shown, the plasticity mechanism is quite different in healthy vs. pathological brains. Moreover, this plasticity brain mechanism could be modulated by education and CR. Specially, cognitive domains as working memory, some executive functions and the visuoconstructive abilities seem to be modulated by education. Therefore, it seems to be crucial, to propose mechanisms of maintenance of a healthy and enriched brain, since it promotes auto-regulatory mechanisms of well-aging.

## Introduction

As human life expectancy increases, maintaining cognitive function has become a target to pursue. It seems that a combination of educational and occupational fulfillment, recreational activities [all aspects included in the concept of cognitive reserve (CR)], and the biological process of aging itself could be the predictor of how to develop cognitive performance as we age.

In recent decades there has been an increasing interest in finding out why some older adults are able to maintain adequate cognitive abilities despite age, while others show clear cognitive decline with advancing age (Wilson et al., 2002). The concept of CR accounts for individual differences in which people with higher CR are able to enlarge the performance on cognitive tasks by recruiting different brain systems and/or using alternative cognitive strategies compared to individuals with lower CR (Stern et al., 2003; Stern, 2009; Tucker and Stern, 2011; Meng and D’Arcy, 2012).

In recent decades it has been demonstrated that age do not affect cognitive functions in the same way nor do they evolve at the same rate throughout life. Processing speed is one of the cognitive abilities that most consistently decline with age (Holdnack et al., 2013; Hong et al., 2015; Tam et al., 2015), even when motor dexterity is statistically controlled (Ebaid et al., 2017). Detriment with age in the case of working memory and inhibition has been also largely demonstrated (Turner and Spreng, 2012; Calso et al., 2016). With advancing age inhibitory mechanisms become increasingly less efficient and no longer prevent irrelevant information from saturating working memory capacity (De Beni et al., 2007). Normal aging seems also to be characterized by a general reduction in cognitive flexibility, defined as attention switching and task shifting. In fact, it has been argued that correlations between cognitive flexibility and primary mental abilities were relatively stable across the adult lifespan, suggesting that individual differences in cognitive flexibility could be responsible for age-related changes in other cognitive abilities (Hülür et al., 2016).

Finally, the extant literature regarding declarative memory indicates that episodic and semantic memory measures are associated with differential aging patterns across lifespan (Nyberg et al., 2012). Thus, several cross-sectional studies have stablished an early onset for episodic memory decline following a linear pattern that begins around the age of 20 or 30 years and results in as much as 1 standard deviation (SD) unit below peak level performance by the age of 60 years and 2 SD units at age 80 years (Schaie, 1994; Nilsson et al., 1997; Verhaeghen and Salthouse, 1997; Park et al., 2002). Longitudinal studies, however, suggest that episode memory decline begins by the age of 60 years followed by an accelerated deterioration (Schaie, 1994; Zelinski and Burnight, 1997). Nevertherless, that discrepancy may be explained by the increase of the educational level in the last decades. On the other hand, semantic memory or knowledge is among the more stable memory systems across the adult lifespan (Park and Reuter-Lorenz, 2009). Young to young–old adults (from age 35 to age 65 years) maintain or even slightly improve their semantic performance, and deficits appear only at very late ages in this domain (Kaufmann, 2001).

Advancing knowledge of influence of CR and educational attainment on aging can be very hopeful since, although the brain will be modified by age, there are numerous opportunities to adapt, restrict, or improve cognitive changes, allowing older adults continue to function independently. Therefore, the purpose of this study is to examine the effect of education and CR in the process of healthy brain aging. We seek to discover whether aging follows similar patterns in different cognitive domains, and how these variables might influence the aging process.

## Materials and Methods

The study was approved by the Ethics Committee of the University of Almeria, and conducted in accordance with Helsinki declaration and Spanish legislation on personal data protection. Participation was voluntary and all subjects gave written consent.

### Subjects

A sample of 140 subjects was recruited from social clubs, entertainment centers, and the University of Almeria’s Center for Adult Education.

The sample was divided into two age groups according to the traditional Spanish retirement age (65 years): adults (aged 36–64 years) and elderly adults (≥65 years). None of them had a history of psychiatric or neurological disorders, drug consumption, or head injury that could potentially affect their cognitive performance. Besides, for all subjects older than 64 years a score of 27 or lower in the *Mini-Examen Cognoscitivo* [Mini-Mental State Examination (Lobo et al., 2002)] was a criterion of exclusion. Following these criteria, three men were excluded and four women (one of them with fibromyalgia diagnosis) under the suspicion of suffering Mild Cognitive Impairment. Additionally, the elderly adults completed the Barthel index (Mahoney and Barthel, 1965). **Table 1** shows the sociodemographic characteristics of the participants.

**Table 1:** Participant demographics, cognitive reserve scale (CRS) scores (León et al., 2014), and descriptive scores for elderly adults.

	Age group (years)	
	Adults (*n* = 98)	Elderly adults (*n* = 42)
Gender Male	34	13
Female	64	29
Age (years)	49.15 (7.18)	71.88 (5.62)
Level of education (years)	13.79 (4.71)	10.12 (5.12)
*Mini-Examen Cognoscitivo*	–	33.7 (1.67)
Barthel index	–	100 (0.0)
CRS	52.98 (10.15)	52.22 (12.58)

### Methods

Subjects completed the cognitive reserve scale (CRS), developed by the authors, that measures the reserve throughout a person’s lifetime by means of taking part in cognitively stimulating activities (reading, playing a musical instrument, collecting things, speak several languages or dialects, traveling, or play sports) (León-Estrada et al., 2017). Each item was completed several times about different age periods: the older the participant, the more times each item had to be completed. A Likert-type scale of 0–4 points was used and the total CRS score was the sum of the mean scores for each item (24 items). The CRS gave scores ranging from 0 to 96, with higher CRS scores indicating more frequent participation. The CRS is available upon request from the authors and additionally, it is available on the Internet: http://www2.ual.es/CognitiveReserveScale/the-cognitive-reserve-scale-crs/.

### Neuropsychological Assessment

A trained psychologist carried out the neuropsychological assessments in several cognitive domains: Working memory and flexibility, Digit Span subtest (backward) (Peña-Casanova et al., 2009c), the Stroop test (Peña-Casanova et al., 2009b), TMT-B (Peña-Casanova et al., 2009c), Controlled Oral Word Association Test (COWAT) (Benton and Hamsher, 1989), and the Corsi Block task (backward) (Peña-Casanova et al., 2009c), visuoconstructive abilities: matrix reasoning and Block Design subtests (Wechsler, 1993), and Rey–Osterrieth Complex Figure Test (ROCF) (quality of copy) (Peña-Casanova et al., 2009a), and declarative memory: Verbal Learning Spanish–Complutense Test TAVEC (Benedet and Alejandre, 1968), sum of the learning slope, short-term recall and delayed-memory, and ROCF short-term recall and delayed-memory. **Table 2** shows the main scores of neuropsychological tests.

**Table 2:** Multivariate analyses of covariance (MANCOVA) classified according to the three functional areas (anterior, posterior and temporal).

	Age group (years)		*p*	$\eta_{p}^{2}$	Covariates			
Functional areas, Mean (*SD*)	Adults (*n* = 98)	Elderly adults (*n* = 42)			Education		CR	
					*p*	*r*	*p*	*r*
**Working memory and flexibility**			0.003	0.126	NS	NS	NS	NS
Digit Span subtest (backward)	10.50 (2.62)	11.50 (1.92)	NS	NS	NS	NS	NS	NS
The Stroop test	9.24 (2.67)	11.00 (2.38)	0.001	0.28	0.049	0.16	0.007	0.23
TMT-B	8.96 (2.55)	10.02 (2.58)	NS	NS	NS	NS	NS	NS
COWAT	42.25 (28.20)	54.22 (27.24)	0.019	0.20	NS	NS	NS	NS
Corsi Block task (backward)	9.32 (2.25)	8.60 (1.34)	0.07	0.15	NS	NS	NS	NS
**Visuoconstructive abilities**			<0.01	0.15	<0.01	0.278	NS	NS
Matrix reasoning subtest	11.45 (2.79)	12.76 (2.99)	0.001	0.28	0.007	0.23	NS	NS
Block Design subtest	10.76 (2.65)	12.44 (2.68)	0.001	0.28	0.001	0.28	NS	NS
ROCF (quality of copy)	6.77 (2.67)	7.85 (2.43)	NS	NS	0.001	0.28	NS	NS
**Declarative memory**			NS	NS	NS	NS	NS	NS
TAVEC sum	0.39 (0.82)	0.44 (1.14)	NS	NS	NS	NS	0.045	0.17
TAVEC short-term recall memory	0.46 (0.95)	0.28 (1.07)	NS	NS	NS	NS	0.014	0.21
TAVEC delayed memory	0.40 (1.03)	0.28 (1.23)	NS	NS	NS	NS	0.036	0.18
ROCF short-term recall	8.90 (5.21)	9.54 (2.65)	NS	NS	NS	NS	NS	NS
ROCF delayed memory	8.31 (2.69)	9.49 (2.86)	NS	NS	0.005	0.24	NS	NS

*Years of formal education and cognitive reserve were entered as covariates for all statistical analyses to control for individual differences. Age was used as independent variable and divided into two age groups: adults (36–64 years) and elderly adults (≥65 years). TMT-B, trail making test part B; COWAT, controlled oral word association test; ROCF, Rey–Osterrieth complex figure test; TAVEC, Verbal Learning Spanish-Complutense Test; CR, cognitive reserve; NS, non-significant.*

In addition, subjects’ IQ score was estimated by Vocabulary subtest (Wechsler, 1993). All the tests are translated and validated in Spanish and the raw scores of all tests were converted to standard scores adjusted for age and educational level (scale scores, *z*-scores, or percentile scores) following Spanish normative studies, except for COWAT, which was standardized following a normative study in an English population (Benton and Hamsher, 1989).

### Statistical Analysis

In the present study, data were analyzed with multivariate analyses of covariance (MANCOVA). A total of three MANCOVAs were conducted separately to investigate the effect of age on the cognitive domains. Three dependent variables were used: the neuropsychological performance in the cognitive domains previously mentioned. Years of formal education and CR were entered as covariates for all statistical analyses to control for individual differences. Age was used as independent variable and divided into two age groups: adults (36–64 years) and elderly adults (≥65 years).

As statistical assumptions underlying the MANCOVAs were not fully met, the estimation of the parameters in the linear model was analyzed using the resampling method of simple bootstrapping with 1000 bootstrap samples. The bootstrap bias-corrected accelerated method was used as a corrective method. Analyses were carried out using statistical package IBM-SPSS 22.0 for Windows (IBM Corp., 2013).

Results with *p* < 0.05 were considered statistically significant. The effect size was obtained by using the partial eta squared ($\eta_{p}^{2}$) and *r* of regression coefficient (*B*).

## Results

In relation to working memory and cognitive flexibility [Digit Span subtest (backward), the Stroop test, TMT-B, COWAT, and the Corsi Block task (backward)], there were significant differences between adults and elderly adults (**Λ**_Wilks_ = 0.874, *F*_5,130_ = 3.735; *p* = 0.003; $\eta_{p}^{2}$ = 0.126). The global effect of the covariates (education and CR) was not statistically significant.

Additionally, estimation of the effect of the independent variables and covariates on the dependent variables showed significant relationships between the Stroop test and age group (*B* = -1.427; *p* = 0.001; *r* = 0.28), education (*B* = -0.098; *p* = 0.049; *r* = 0.16), and CR (*B* = 0.49; *p* = 0.007; *r* = 0.23), as well as between COWAT and age group (*B* = -13.70; *p* = 0.019; *r* = 0.20), and the Corsi Block task (backward) and age group (*B* = 0.98; *p* = 0.07; *r* = 0.15) (**Table 2**).

In the case of the visuoconstructive abilities [the Matrix Reasoning, Block Design, and ROCF (quality of copy)], there were statistically significant differences between adults and elderly adults (**Λ**_Wilks_ = 0.847, *F*_3,130_ = 7,817; *p* < 0.01; $\eta_{p}^{2}$ = 0.15). The effect of education was significant (**Λ**_Wilks_ = 0.722, *F*_3,130_ = 16.645; *p* < 0.01; $\eta_{p}^{2}$ = 0.278), but no significant effect from CR.

The estimation of the effect of the independent variables and covariates on the dependent variables using simple bootstrapping disclosed significant relationships between the Matrix Reasoning subtest and age group (*B* = -1.75; *p* = 0.001; *r* = 0.28) and education (*B* = 0.14; *p* = 0.007; *r* = 0.23); the Block Design subtest and age group (*B* = -2.45; *p* = 0.001; *r* = 0.28) and education (*B* = -0.187; *p* = 0.001; *r* = 0.28), and the ROCF (copy) and education (*B* = -0.231; *p* = 0.001; *r* = 0.28) (**Table 2**).

Regarding the memory tests (TAVEC sum, TAVEC short-term recall, TAVEC delayed recall, and ROCF short-term recall and long-term recall), there were no significant differences between adults and elderly adults. Besides, the effect of the two covariates (education and CR) was not statistically significant.

Finally, estimation of the effect of the independent variables and covariates on the dependent variables using simple bootstrapping revealed significant relationships between TAVEC-sum and CR (*B* = 0.017; *p* = 0.045; *r* = 0.17), TAVEC short-term recall and CR (*B* = 0.021; *p* = 0.014; *r* = 0.21), TAVEC delayed recall and CR (*B* = 0.019; *p* = 0.036; *r* = 0.18), and ROCF delayed recall and education (*B* = -0.149; *p* = 0.005; *r* = 0.24) (**Table 2**).

## Discussion

In the present study, a functional analysis of brain has been performed using neuropsychological tests in two age-differentiated populations. The aim was to assess the evolution of cognitive changes across the life cycle and their relation to educational level and CR.

Our results, generally speaking, show a large influence of aging on inhibition, flexibility, working memory, and visuoperceptive functions, although there is also an important contribution of education in these domains. However, surprisingly, the same pattern was not found in declarative memory tasks where the effect of education and CR but not of aging could be verified. It seems clear that at least in a healthy population, an enriched environment allows us to face the tasks of flexibility, updating, monitoring, or memory as we get old.

Given our results in cognitive domains related to the frontal region, the data in the flexibility, inhibition, and spatial working memory tests have shown an influence of aging, while in inhibition tasks there is also a significant influence of education and reservation.

In the case of frontal areas, it is now well-established that normal aging is associated with cognitive decline (Craik and Salthouse, 2000; Rypma and D’Esposito, 2000; Kennedy et al., 2009), particularly in tasks involving executive functions (Salthouse et al., 2003; Podell et al., 2012). However, all executive functions do not decline in the same way with advanced age (Collette and Salmon, 2014). A previous study from our group (Roldán-Tapia et al., 2012) reported that the “performance of functions related to the dorsolateral prefrontal cortex (PFC) (verbal fluency, behavioral spontaneity, reasoning, divided and complex attention, and working memory) was associated with aging.” Or, for example, regarding to shifting abilities, older people would show difficulties to maintain and to manipulate two mental plans but not to alternate between both of them (Kray et al., 2004). However, these results are not consistent with Schaie (1958, 1994) and Stawski et al. (2013) works, that show a stable performed in flexibility during lifespan (although it is true that they did an intra-subjects analyses).

In addition, numerous researches have shown how CR and education exert a protective effect on the decline associated with age in executive functions (Ardila et al., 2000; Opdebeeck et al., 2016).

On the other hand, the effect of aging on the visuoconstructive domains has also been demonstrated earlier, mainly in face recognition, mental rotation, and visuospatial abilities (Adduri and Marotta, 2009; Iachini et al., 2009; Daniel and Bentin, 2012). It seems that the occipital decrement and sensory deficits are consistent with perceptual processing declines as a function of aging (Zhuravleva et al., 2014).

The relationship between cognitive decline that occurs in the elderly, and age-related changes that take place in brain morphology and functioning, is still being documented.

Virtually no area of the brain is fully spared from the effects of aging, although certain brain systems seem to be particularly vulnerable to aging effects, which takes place earlier and in greater degree (Raz, 2000). From a system-vulnerability approach, the deterioration associated to age would be the collection of repercussions during a life. Brain structure and function are modified in a multidimensional way, which could affect different aspects such as structural integrity, functional activity, and connectivity, as a result of continuous interactions between endogenous and exogenous factors (Khachaturian, 2011; Jagust, 2013). The areas found to be most vulnerable to normal age changes are the PFC and the medial temporal cortex (Raz, 2000, 2004; Buckner, 2004; Hedden and Gabrieli, 2004).

But perhaps the most relevant fact is the influence of education on complex visuoconstructive tasks such as spatial reasoning, visuoconstruction, and the grafomotor integration of a complex design. Our data are in line with previous studies such as that of Ardila et al. (2000) which points out the importance of education for the execution of cognitive processes, especially in older people and even seems to play a relevant role in elderly “illiterates.”

The results of the present study are also supported by data from other studies. For example, Kim et al. (2015) demonstrated a protective effect of education on the cortical thinning in cognitive normal older individuals. According to the authors, this protective effect could be achieved by increasing resistance to structural loss from aging.

In memory domains, our results point to the idea of certain stability across different age periods. This result is not new; other studies have supported the idea of maintenance of different types of memory (semantic memory) in healthy subjects (Park and Reuter-Lorenz, 2009). For example, the perirhinal cortex appears to undergo little age-related atrophy (Insausti et al., 1998). Also, performance in older adults seem be associated with the recruitment of additional brain regions in the medial temporal lobe and in frontal regions (Cabeza, 2001; Maguire and Frith, 2003; Giovanello and Schacter, 2012). It is no less true, however, that a group of studies have shown hippocampal degeneration (Raz, 2004; Raz and Rodrigue, 2006) or a reduction in BOLD signal in the medial temporal lobe (Maguire and Frith, 2003).

While in the case of the working memory, flexibility, and visuoconstructive domains, we do not find any relevant influence from CR in the aging process, the same cannot be said for mnesic processes. Structural MRI studies in healthy aging consistently reported positive associations between CR and increased gray and white matter volumes in associative frontal and temporoparietal cortices, as well as reduced mean diffusivity in the bilateral hippocampus (Akbaraly et al., 2009; Marioni et al., 2012). Declarative memory is greatly influenced by education, primarily, and also by CR, as well in short-term memory and delayed recall, as using visual and verbal stimuli. Valenzuela et al. (2008) found that hippocampal decline occurs more slowly in normal aging when performing cognitive challenging life.

It has also been shown that greater CR contributes to delay or attenuate pathological changes such as those that occur in Alzheimer’s disease (Carnero-Pardo and Del Ser, 2007), vascular injury (Dufouil et al., 2003; Elkins et al., 2006), Parkinson’s disease (Glatt et al., 1996), traumatic brain injury (Kesler et al., 2003), neuropsychiatric disorders (Barnett et al., 2006), and multiple sclerosis (Sumowski et al., 2009); greater CR may even prevent accumulation of amyloid plaque (Landau et al., 2012). Observations using cerebral blood flow (CBF) have found that Alzheimer’s disease patients with higher education have a lower resting rCBF (Stern et al., 1992).

In individuals with AD and mild cognitive impairment, higher education and occupation (as proxies of CR) are correlated to more severe hypometabolism in temporoparietal areas (memory and visual abilities) and in the precuneus (Garibotto et al., 2008). Moreover, they are associated with an increased metabolism in the dorsolateral PFC (working memory and flexibility), suggesting a compensatory mechanism against AD-related cerebral neurodegeneration (Grady et al., 1994). A review paper calculated that a higher CR reduced the chances of suffer dementia by 46% (Valenzuela and Sachdev, 2005). However, even higher CR cannot maintain functions when pathology turns very severe.

Could it be that education affects cognitive abilities (processing speed, working memory, verbal fluency, or verbal episodic memory) differently?

Maybe, in demanding memory or complex visuoperceptive tasks, the influence of variables such as reserve and education is more marked in the case of healthy aging or even more visible in the case of pathological aging than in other cognitive domains. The domain of memory could be more sensitive to the effect of training, education, and lifestyle.

Our results show that, in healthy people, if we keep level of education and CR constant, the only variable that can influence the neuropsychological outcome in flexibility, working memory, and visuoconstructive domain, seems to be aging. Nevertheless, our data are collected from subjects who were healthy and not very advanced in age. It may be that the possible effect of CR depends on the baseline of normal versus pathological brain conditions. From this point of view Hayden et al. (2011) defined “reserve” as “the difference between cognitive performance as predicted by an individual’s brain pathology and that individual’s observed cognitive performance.” Thus, people whose measured cognitive performance is better than predicted by their pathology have high reserve, whereas those who perform worse than predicted have low reserve.

Regarding the age of our study participants, a 15-year longitudinal study among elderly Catholic clergy members who were participating in the religious Orders (healthy aging) showed a typical profile of extraordinarily slow deterioration of cognitive functions, which last for decades. These findings suggest that cognitive changes associated with aging may be minimal when the process is not associated with a neurodegenerative disease (Christensen et al., 1994; Hayden et al., 2011; Reed et al., 2011).

Both our results and those from morphological and cognitive studies lead us to the idea that “brain aging” is an interactive and synergistic process in which several variables play an important role.

For example, the health condition versus pathology, the genetic load, and the influence of an enriched environment (CR and education). A good method to further investigate this interactive process is the use of brain mapping approaches to complete information obtained through the neuropsychological assessment of cognitive domains (Agis and Hillis, 2016) such as the ones described in the present study (working memory, flexibility, visuoconstructive functions, and declarative memory).

Additionally, the new developments of neuropsychological brain mapping batteries seem to contribute to enhance clinical diagnosis, pre-surgical mapping, and follow-up (Karakas et al., 2013). Hence, advances in the knowledge of neural substrates involved in cognitive tests and revealed by the interpretation of human brain mapping studies represent a strong resource to increase the comprehension of brain and how age and different levels of education or CR might affect cognitive vulnerability.

### Limitations

Limitations of the current study are the size of the sample and the lack of inclusion of people with presumably low reserve (without access to education or social club). The present sample seem likely to have a higher than average CR, but the CRS includes a high variety of different activities that are not limited exclusively to the centers where the participants were recruited. Besides, in the elderly adults, the SD related to the years of formal education reflects variability among them (from just 5–15 years of formal education). However, future studies should include an older sample, as well as specific experimental tasks, to help discern the real influence of CR in healthy aging populations.

As a final conclusion, regarding the importance of CR in the domain of memory, its limited effect on fluency, divided attention, interference, spatial reasoning, and visuospatial tasks may be due to the brain’s own compensation mechanism, which under healthy conditions, makes it independent of education or CR. It is the effect of CR could be quite different depending of the brain status: pathological or healthy.

## Author Contributions

Conceived and designed the experiments: MR-T, JG-G, and IL. Performed the experiments: IL. Analyzed the data: JG-G and IL. Wrote the paper: RC, MR-T, and JG-G.

## Conflict of Interest Statement

The authors declare that the research was conducted in the absence of any commercial or financial relationships that could be construed as a potential conflict of interest.
